# Monthly Summary

**Published:** 1877-12

**Authors:** 


### MONTHLY SUMMARY.

*Status of English Dentistry.*—The following epitome of the status of English Dentistry, is given by the *Lancet.* Dividing the profession into two classes, the "Dental Surgeons" and the "Dentists," the writer says:

"The dental surgeons, as a body desire to elevate their profession, and to keep it at as high a position as possible, to claim for it the same rank and the same educational requirements as ophthalmic, orthopædic, or aural surgery; and, while accepting and fully recognizing the value of licentiateship, aspire to and encourage full surgical culture and diploma. On the other hand, the dentists consider the L. D. S., a sufficient and the only desirable qualification, (though a large proportion of their party have not even taken this modicum of title,) and indirectly discourage the membership or fellowship of the College, and to support which doctrine they now seek to obtain from the Legislature a separate registration, to be shielded beneath the pages of the Medical Register under a new act which they have sketched out."

---

*Simple Method of Testing the Purity of Chloroform.*—Dr. Lueke, of Strasbourg, gives the following simple method of testing the purity of chloroform: Immerse a small piece of thin white blotting-paper into the chloroform, and then let it dry in the air. As soon as all the chloroform has evaporated, the paper will not present the least smell if the chloroform is pure. If there is any acid smell perceptible, it indicates the presence of butyric acid in the chloroform, and as a rule has the strong characteristic odor of that substance.—*New Remedies.*

---

*Lionel S. Beale.*—The *Chemist and Druggist.* calls Dr. Lionel S. Beale, "one of the few scientific men whose faith is broader than the revelations of his microscope."

*Death from Transfusion.*—A man died in Liverpool, England lately, from having had his blood transfused into another man who was ill. He went on all well for a day or two afterwards. He then became ill, got gradually weaker and died from erysipelas. The deceased was a man of full habit, and was occasionally given to drinking. The surgeon who performed the operation, before doing so, made particular inquiries from the deceased as to his habits and state of health, and his answers were satisfactory. At the inquest medical evidence was to the effect that the operation had been skillfully performed. The jury returned a verdict of "death by misadventure," but they were also of opinion that sufficient inquiry was not made by the medical men who made the operation as to the deceased's habits and physical condition, and that he was not sufficiently cautioned as to the risk he was running.—*Druggists Circular.*

---

*External Use of Chloral Hydrate*—A writer in the *Canada Medical Record* recommends a solution of chloral hydrate in water, from three to five grains to the ounce, as a substitute for carbolic acid for external use. Its effect on ulcers is prompt and admirable, and a case of eczema was successfully treated by it. In cases of amputation, where the surfaces took on unhealthy action, the solution changed their character and removed the difficulty in twenty-four hours. It removes fetor, is clean and neat, does not stain, stimulates granulations without destroying them.

Chloral hydrate has also been used in Germany as an antiseptic application in cancer of uterus. A strong solution—one part to twelve of water—is applied by means of cotton. Dr. Goodell of Philadelphia, also speaks highly of it as a purifying and deodorizing agent.

---

*American Medical Diplomas Abroad.*—It would appear that our English cousins are never to learn what constitutes a true medical diploma in this country. The name American diploma is so constantly associated in their minds with the bogus certificates which are sold by *quasi* institutions here, that it is difficult for them to understand that we have any other legal guarantees for medical education. The facts of this abominable traffic have been brought to the notice of the profession across the water time and time again, and it should be pretty generally understood that the quacks who settle in London, and who claim to be "American" physicians, are no more recognized here than they are there. The profession here has done its best to check the bogus diploma trade, and to a certain extent it has succeeded. It should not be held accountable for what it cannot control; and further, it is hardly fair to class what are really well-educated medical men here with a class whom even our English brethren acknowledge as transparent frauds.

Every little while a new case comes into the London courts under the sensational caption of "Another American Physician in Trouble," and we read the account to find that a "Dr." Hamilton or "Dr." Jones has been guilty of unlawful conduct in assuming a spurious title, etc., etc.; and then, in the course of the comments upon the value of American diplomas and the laxity of requiremeuts for a medical degree, the usually absurd questions are asked if there be such a recognized medical school in the United States as the Metropolitan Medical College, the School of Medicine of New York, the Medical University of Philadelphia, *et id omne genus.* We protest that we have had enough of this, and think it about time that our critics should inform themselves of the true state of medical affairs in this country before indulging in further remarks—*Med. Record.*

---

*The Dangers of Ether.*—It has always seemed to us the height of folly to declare there could be no danger in any anæsthetic. The lesson taught by the late death from nitrous oxide has, it is to be hoped, been well learned, and we shall in future hear less of the absolute safety of any agent capable of depriving a person of all sensation. Some cases in which ether has been followed by alarming symptoms have lately been recorded. They have been termed syncope, but the word is not appropriate, as the heart continued to beat after respiration ceased. This is what should have been anticipated. When death is produced by ether the animal's heart continues to beat long after the arrest of respiration. The pulse is quickened by ether and maintains its force through a long state of anæsthesia. In these facts lies the safety of ether. But it should never be forgotten that there is danger at a certain state, and the danger is from the side of the respiration, which at length ceases. Stertorous breathing proceeds from paresis of the muscles of the palate, and should lead to the ether being suspended. So respiration growing more and more shallow and less frequent is a warning, and should not be overlooked. It is very rare that the heart fails—perhaps never. Pallor is rare, too, and should excite attention if it occur. But, we repeat, the danger of ether is from the side of respiration, that of chloroform from the heart and this fact goes far to explain their relative safety. In chloroform narcosis the danger is much more sudden. Ether gives warning.—*The Doctor.*

*Making Rubber Stamps.*—Have a vulcanizing apparatus with a thermometer and a lamp under it, such as dentists use ; have an iron printing frame in which you lock up the type for all the names which you wish to reproduce in rubber and of such a size that the plaster mold made from it can be placed inside the vulcanizer. This mold is made like an ordinary stereotype mold, by first oiling the type and then pouring the plaster over it ; when set, take it off carefully, and do not let it dry but proceed at once by placing on top of the mold a piece of sheet rubber [not pure rubber, as this is too sticky, but vulcanized, and mingled with sulphur soap stone.] Then have two iron plates, one for placing on top of the sheet-rubber and one below the plaster mold, and which by proper screws can be pressed together and squeeze the rubber on the mold. Back up the rubber with few sheets of paper, so as to prevent it from sticking at the back of the iron plate. After screwing down sufficiently, immerse the mold and rubber in the water in the vulcanizer, screw the cap on, and heat to 300° Fah., then let it cool, open the vulcanizer, take out the mold and rubber, and remove the rubber carefully. This will be easily done if you have put the mold, while still wet, in the vulcanizer. Cut up the rubber so as to separate the various names, glue them to handles, and your rubber hand-stamps are ready. This is the regular method, and if you are not satisfied with the impression given, it is the fault of the manipulation. We have such stamps, which give as clear an impression as can possibly be desired—as clear as metal type. But in printing with it you must apply a slight pressure only ; the best rubber type can be made to give bad impressions by defective inking and rough manipulation in printing.—*Manufacturer and Builder.*

---

*Chloral in Tetanus.*—At a late meeting of the Paris surgical Society, M. Gueniot read a report on a case communicated by M. Ganiez. The cause of the tetanic attack in this case was rare as the disease became developed twenty-two days after an amputation of the breast. Large doses of chloral, twenty-five grammes (seven-eighths of an ounce,) were given every twenty-four hours. On the fourth day the effect was so manifest that a speedy cure was anticipated ; but the patient was not perfectly well before five weeks. About three and a half ounces of chloral were given altogether.—*Med. Examiner.*

